# Genetic polymorphisms in *CYP1A1*, *CYP1B1* and *COMT* genes in Greenlandic Inuit and Europeans

**DOI:** 10.3402/ijch.v72i0.21113

**Published:** 2013-06-17

**Authors:** Mandana Ghisari, Manhai Long, Eva C. Bonefeld-Jørgensen

**Affiliations:** Centre for Arctic Health and Unit of Cellular and Molecular Toxicology, Department of Public Health, Aarhus University, Denmark

**Keywords:** Inuit, genetic polymorphism, cytochrome P450, COMT, persistent organic pollutants, PCBs

## Abstract

**Background:**

The Indigenous Arctic population is of Asian descent, and their genetic background is different from the Caucasian populations. Relatively little is known about the specific genetic polymorphisms in genes involved in the activation and detoxification mechanisms of environmental contaminants in Inuit and its relation to health risk. The Greenlandic Inuit are highly exposed to legacy persistent organic pollutants (POPs) such as polychlorinated biphenyls (PCBs) and organochlorine pesticides (OCPs), and an elucidation of gene–environment interactions in relation to health risks is needed.

**Objectives:**

The aim of this study was to determine and compare the genotype and allele frequencies of the cytochrome P450 *CYP1A1* Ile462Val (rs1048943), *CYP1B1* Leu432Val (rs1056836) and catechol-*O*-methyltransferase *COMT* Val158Met (rs4680) in Greenlandic Inuit (n=254) and Europeans (n=262) and explore the possible relation between the genotypes and serum levels of POPs.

**Results:**

The genotype and allele frequency distributions of the three genetic polymorphisms differed significantly between the Inuit and Europeans. For Inuit, the genotype distribution was more similar to those reported for Asian populations. We observed a significant difference in serum polychlorinated biphenyl (CB-153) and the pesticide 1,1-dichloro-2,2-bis(*p*-chlorophenyl)-ethylene (*p,p*′-DDE) levels between Inuit and Europeans, and for Inuit also associations between the POP levels and genotypes for *CYP1A1*, *CYP1B1* and *COMT*.

**Conclusion:**

Our data provide new information on gene polymorphisms in Greenlandic Inuit that might support evaluation of susceptibility to environmental contaminants and warrant further studies.

Genetic polymorphisms including single nucleotide polymorphisms (SNPs) that affect xenobiotic metabolism may modulate the individual susceptibility to environmental contaminant exposures and the risk of developing cancers (1). The genetic background of the Greenlandic Inuit is different from the Caucasian population. Previous DNA typing of mitochondrial DNA of Inuit (2) has supported the genetic link of Inuit with East Asian populations. To date, relatively little is known about the specific genetic polymorphisms in the Inuit population in relation to environmental contaminants and health risk. Greenlandic Inuit are highly exposed to persistent organic pollutants (POPs) such as polychlorinated biphenyls (PCBs) and organochlorine pesticides (OCPs) mainly through consumption of their traditional marine food (3). The levels of bioaccumulated POPs in blood of Europeans and Inuit differ primarily because of differences in diet and lifestyle (4,5). The genetic backgrounds are likely to affect the toxicity of contaminants and susceptibility to an array of diseases (6).

Phase I xenobiotic metabolizing enzymes, such as cytochrome P450 (e.g. CYP1A1 and CYP1B1), play important physiological roles in the detoxification of xenobiotics and the biosynthesis of endogenous steroid hormones [reviewed by Nebert and Dalton (7)]. These enzymes metabolically activate procarcinogens to reactive electrophilic forms, reactive oxygen species (ROS), which can damage DNA if they are not detoxified by phase II enzymes [e.g. catechol-*O*-methyltransferase (COMT)]. Hence, an imbalance between phase I and phase II enzymes may increase ROS production leading to oxidative stress. Inter-individual and inter-ethnic variations in the metabolism of environmental agents and the potential susceptibility to carcinogenicity can be influenced by genetic polymorphisms of many enzymes involved in these processes (8).

*CYP1A1* encodes aryl hydrocarbon hydrolase (AHH), an enzyme involved in the production of reactive epoxide intermediates from environmental contaminants such as polycyclic aromatic hydrocarbons (PAHs), polyhalogenated aromatic hydrocarbons (PHAHs) (e.g. dioxin-like compounds and PCBs) and steroid hormones (7,9) that might increase the risk of oxidative stress and cancer. In addition, CYP1A1 also catalyzes the 2-hydroxylation of 17β-estradiol (E2) into 2-hydroxyestradiol (2-OH-E2).

*CYP1A1* gene expression is induced by PAHs, dioxins/furans and dioxin-like PCB congeners (DL-PCBs) mediated via the aryl hydrocarbon receptor (AhR) (10,11). Animal experiments have revealed a protective role of CYP1A1 induction in PAH toxicity as *Cyp1A1*-null mice were more susceptible to benzo[*a*]pyrene (BaP)-induced toxicities, indicating inducible *Cyp1A1* functions in the detoxification and protection against oral BaP (12). This result was not expected because *Cyp1A1* can also metabolically activate BaP as revealed *in vitro*.

Several SNPs have been identified in *CYP1A1*, some of which lead to a more highly inducible AHH activity (9). One *CYP1A1* SNP includes the A to G transition at position 4,889 in exon 7 resulting in a change from an isoleucine to valine amino acid (Ile→Val) at codon 462 (13). This variant is significantly associated with *CYP1A1* inducibility and higher AHH enzyme activity (9,14) that might cause higher rates of carcinogen activation. Hence, individuals with the variant *CYP1A1* gene (Val) and thus higher inducibility and AHH enzymatic activity may be more susceptible to xenobiotic carcinogens and health risk. In Caucasians, the *CYP1A1* Val/Val genotype was suggested to be associated with a higher risk of breast cancer (15), whereas in Chinese and Japanese this amino acid substitution was associated with other types of cancer, such as lung cancer (16). The frequency of the variant Val allele differs between Caucasian and Asian and is about 0.052 and 0.228, respectively (17).

The *CYP1B1* gene encodes another phase I enzyme that metabolically activates PAHs, heterocyclic and aryl amines, and nitroaromatic hydrocarbons (18). CYP1B1 is also involved in the oxidative metabolism of estrogens and preferentially catalyzes the hydroxylation of E2 to the catechol estrogen metabolite, 4-hydroxyestradiol (4-OH-E2) (19), a very estrogenic and potential genotoxic metabolite playing a role in carcinogenesis. As for *CYP1A1*, the expression of *CYP1B1* can be induced by several AhR agonists, such as dioxins, PAHs and DL-PCBs (11). Mice lacking expression of *CYP1B1* showed normal development and had no observable phenotype, demonstrating that CYP1B1 is not required for mouse development (20), and CYP1B1-null mice were protected against PAH-induced tumours in most tissues suggesting that CYP1B1 is required for metabolic activation and thus the carcinogenic potential of PAHs (20).

Four sense polymorphisms of the *CYP1B1* gene have been found of which an important single nucleotide transversion at codon 432 in exon 3 (C→G), leads to an amino acid substitution of leucine to valine (Leu→Val), which increases the 4-OH activity of CYP1B1 3-fold (21–23). However, the catalytic activity of CYP1B1 to form reactive metabolites from procarcinogens (e.g. PAHs and heterocyclic aryl amines) was slightly higher in the wt Leu432 (21).

Considering its role in procarcinogen activation and oxidative metabolism of estrogens, there have been several reports on the role of *CYP1B1* polymorphisms and a variety of estrogen mediated diseases, for example, breast and prostate cancer and controversial results have been reported (24,25). The frequency of the variant *CYP1B1* Val allele is reported to be 0.43 in Caucasians and 0.23 in Asians (24).

A major inactivation step for 2-OH-E2 and 4-OH-E2 catechol estrogens is the conversion to their non-genotoxic methoxy derivatives (2-MeOE2, 2-OH-3-MeOE2, and 4-MeOE2) by the phase II enzyme COMT. Generally, COMT methylates a wide range of catechol substrates, including catecholamines (e.g. dopamine) and catecholestrogens (26). Methylated catechol estrogens have little or no binding affinities for estrogen receptors, and they do not exert estrogenic effect on target tissues (27). In fact, 2-methoxyestradiol (2-MeOE2) inhibits angiogenesis and is a strong inhibitor of tumour cell proliferation (28).

In the *COMT* gene, a single G to A base pair change results in an amino acid change from valine to methionine (Val→Met) at codon 108 of the soluble form of COMT and codon 158 of the membrane-bound form of COMT. This amino acid change has been associated with a 3- to 4-fold decrease in enzyme activity *in vitro* (29,30), and designated as the COMT-L (low activity) variant in contrast to the wild-type COMT-H (high activity). The alleles are codominant, and the Val/Met heterozygotes have intermediate levels of COMT activity. Individuals carrying the variant Met allele are hypothesized to have a decreased ability to form the anti-tumour 2-MeOE2, causing an increased accumulation of the reactive catechol estrogen intermediates and thus facilitating the development of estrogen-induced tumours such as breast cancer. Epidemiological study data on the influence of the COMT-L variant (Met/Met) with respect to human cancers such as breast (31,32), endometrial and prostate carcinoma (33) are controversial. In the European population, a near-equal frequency of the two alleles exists, while the wild type Val allele is much more common in the Asian population (34). The variant Met allele frequency for Caucasian and Asian is 0.53 and 0.28, respectively (35,36).

Interethnic differences in the allele distribution in xenobiotic metabolizing genes have been described between Caucasians and many other populations (17). However, almost no data are available for the Inuit populations. The aim of this study was to determine the allele frequencies of *CYP1A1* (Ile462Val), *CYP1B1* (Leu432Val) and *COMT* (Val158Met) in Greenlandic Inuit and Europeans. Moreover, we aimed to analyze whether these polymorphisms are associated with the serum POP levels in order to provide an insight into possible ethnic differences in metabolism and susceptibility to environmental contaminants.

## Methods

### Study population

This study was a part of a fertility cross-sectional study, INUENDO, conducted on Greenlandic and 3 European populations (5), the International Polar Year and Arctic Monitoring and Assessment Programme (AMAP) studies (4,37). The study population consisted of Greenlandic Inuit (64 males and 190 females), defined as being born and having grandparents born in Greenland. The participants were from the following districts in Greenland: 214 from West (Sisimiut and Nuuk); 27 from East (Tasiilaq); 7 from South (Narsaq, Nanortalik, Qaqortog) and 6 from Midwest (Assiaat, Upernavik). The sampling period was 2000–2003 (female) and 2002–2004 (male).

Furthermore, 262 males from 3 European countries [Warsaw, Poland (n=80); Kharkiv, Ukraine (n=84) and Sweden (n=98)] from the established Inuendo cohort (http://inuendo.dk), collected from 2002–2004 (5), were included in the study. Unlike the other European participants, the Swedish participants were from a fishermen's population living on the east coast of the Baltic Sea.

Blood sample collection and the details about the inclusion/exclusion criteria for the participants have been described elsewhere (5,37,38).

The study was approved by the local Ethics Committees of all participating populations.

### DNA isolation and genotyping

Genomic DNA was extracted from whole blood using the QIAamp DNA mini Kit (Qiagen) according to the manufacturer's recommendations. Genotypes for the *CYP1A1* Ile462Val (rs1048943) and *CYP1B1* Leu432Val (rs1056836) and *COMT* Val158Met (rs4680) polymorphisms were determined using the TaqMan Drug Metabolism Genotyping Assays C_25624888_50, C_3099976_30 and C_25746809_50, respectively, using protocols described by the manufacturer (Applied Biosystems, ABI). Table I shows the specifications of these polymorphisms. The assay was done in a 12 µL reaction volume containing 1× TaqMan Drug Metabolism Genotyping Assay Mix (containing premixed PCR primers and MGB probes), TaqMan Universal PCR Master mix and 10–15 ng genomic DNA. Allele discrimination analysis was performed on the ABI Prism 7,000 Sequence Detection System.

**Table I:** Characteristics of the studied gene polymorphisms

						Activity	
						
Gene	Chromo-some	SNP	Reference SNP	TaqMan assay	Main function	Low	High
*CYP1A1*	15q22–24	Ile462Val	rs1048943	C_25624888_50	Carcinogen activation; 2-hydroxylation of E2	Ile (wt)	Val (var)
*CYP1B1*	2p22–21	Leu432Val	rs1056836	C_3099976_30	Carcinogen activation; 4-hydroxylation of E2	Leu^a^ (wt)	Val^b^ (var)
*COMT*	22q11.2	Val158Met	rs4680	C_25746809_50	Estradiol catabolism	Met (var)	Val (wt)

wt: wild-type; var: variant.

a Leu432 has the lowest 4-OH activity; but higher carcinogenic activity (21)

b 432val variant has the highest 4-OH activity; but lower carcinogenic activity (21).

Positive controls consisting of DNA representing wt/wt, wt/variant and variant/variant genotypes and negative controls (no DNA) were included in each assay. The samples were genotyped in duplicate, and when discrepancies were discovered, the genotyping were repeated for these samples (for less than 2% of the samples).

Not all SNPs were successfully genotyped in every sample (due to low DNA quality). The SNPs in *CYP1A1, CYP1B1* and *COMT* were genotyped for 94%, 99% and 99% of the total samples, respectively. Thus, the numbers reported for specific polymorphisms may vary from the total.

### Determination of CB-153 and p,p′-DDE in serum

Serum concentrations of 2,2′,4,4′,5,5′-hexachlorobiphenyl (CB-153) and 1,1-dichloro-2,2-bis (p-chlorophenyl)-ethylene (*p,p′*-DDE) were determined using solid phase extraction (SPE) and on-column degradation of lipids followed by analysis with gas chromatography mass spectrometry as described (5,37). Levels of detection and coefficients of variation have been described in detail previously (5,37), and the laboratories are certificated laboratories and participate in inter-comparison programs. CB-153 and *p,p′*-DDE levels were adjusted for serum lipids and expressed as ng/g lipid.

### Statistical analysis

All statistical analyses were performed using SPSS 19.0 statistical software (SPSS Inc., Chicago, IL, USA). A two-sided p≤0.05 was considered statistically significant in the analysis.

A χ^2^-test was used to determine whether the genotypes were in Hardy–Weinberg equilibrium. Allele frequency differences between Inuit and Europeans were estimated by the Fisher's exact test.

The natural logarithmic transformed variables improved the normality and homogeneity of variance, and thus the comparison analysis was performed on the ln-transformed data. An independent student *t*-test was used to compare the graphical variables [age, body mass index (BMI)] and chemical variables (CB-153 and *p,p′*-DDE) between Inuit and European.

Analysis of covariance (ANCOVA) was used to compare mean serum levels of CB-153 and *p,p′*-DDE between subjects with different genotypes (for each polymorphism) adjusting for potential covariates (sex, age and smoking and place of residence). Based on our earlier studies age, sex, country, and smoking status were potential determinants of serum POP bioaccumulation (5,39), and therefore the model was adjusted for these factors. Adjustment for BMI did not change the results, and therefore this was not included. The area of residence was categorized into regions for Greenlandic Inuit (East, South, West and Midwest) and for Europeans into countries (Poland, Ukraine and Sweden).

If differences were observed, multiple comparison ad hoc tests were performed using the Bonferroni pair wise multiple comparison test for the variables with equal variance (p>0.05 in Levene's test) and Dunnett's T3 test for the variables with an unequal variance (p≤0.05 in Levene's test). Bonferroni adjusts the significance level for multiple comparisons. The homogeneity of variance was tested by Levene's test.

Linear regression analysis was also used to study the association between the *CYP1A1, CYP1B1* and *COMT* SNPs and serum levels of CB-153 and *p,p′*-DDE in the two ethnic populations (gene-dose effect). The genotype for each SNP was given ordinal value as described above (1, 2, and 3; one assigned to each genotype). The model was adjusted for the above-mentioned covariates.

## Results

In total, 254 Greenlandic Inuit and 262 Europeans (Sweden, Poland and Ukraine) were included in the study. The general characteristics of the study population are given in Table II. Significant differences were found in mean age, BMI and current smoking status between the Inuit and the European study groups, where the Inuit group was older, had higher BMI and included more smokers. However, no difference in BMI was found between the men in the two ethnic groups, but a higher BMI was observed for Inuit women.

**Table II:** Characteristics of the study population

		Europeans (Men)					Greenlandic Inuit (Men and women)					
			
		Poland	Sweden	Ukraine	All	p^a^	Men	Women	All	p^b^	p^c^	p^d^
Total	n	80	98	84	262		64	190	254			
Age (years)	Median	29.3	46.6	25.1	30.9	<0.0001	29.5	55.0	52.0	<0.0001	<0.0001	<0.0001
	Min	23.5	23.8	16.2	16.2		18.3	29.0	18.3			
	Max	46.3	67.5	45.3	67.5		43.2	80.0	80.0			
BMI (kg/m^2^)	Median	25.1	26.0	23.4	25.2	<0.0001	25.7	26.9	25.7	0.098	0.002	0.583
	Min	18.9	21.5	18.9	18.9		11.6	14.4	11.6			
	Max	38.1	37.2	35.9	38.1		57.8	43.4	57.8			
Smoking (current)	n (Yes/No)	42/37	57/39	67/16	166/92	0.001	53/10	116/44	169/54	0.083	0.007	0.002

a p Value for comparison among the European countries

b p value for comparison between the sexes among Inuit

c p value for comparison between the Inuit and Europeans

d p value for comparison between Inuit and Europeans, men only.

The studied populations were in Hardy-Weinberg equilibrium (HWE) for all of the tested polymorphisms (*CYP1A1* (Ile462Val), *CYP1B1* (Leu432Val) and *COMT* (Val158Met)). The Greenlandic study population included both sexes, but no gender genotype differences were observed for any of the investigated SNPs (Supplementary Table I).

### Allele and genotype frequencies in CYP1A1, CYP1B1 and COMT gene

In general, comparison of the genotype and allele frequencies between the European countries showed no differences, except the Leu/Leu wt genotype frequencies for *CYP1B1* in Sweden were significantly (p=0.040) lower than Poland. Thus, the data from the three European countries were pooled and subsequently the genotypes between the pooled Inuit and pooled Europeans were compared.

The allele frequencies of *CYP1A1, CYP1B1* and *COMT* polymorphisms differed significantly between the Inuit and Europeans (Table III).

**Table III:** Genotype and allele frequencies of *CYP1A1*, *CYP1B1* and *COMT* polymorphisms in Greenlandic Inuit and Europeans

		Genotypes (%)				Allele		
			
		n	Ile/Ile	Ile/Val	Val/Val	Ile	Val	p^a^
*CYP1A1* (Ile462Val)	Inuit	253	73 (29)	127 (50)	53 (21)	0.540	0.460	
	Europeans	229	203 (89)	26 (11)	0	0.943	0.057	0.0001
			Leu/Leu	Leu/Val	Val/Val	Leu	Val	
*CYP1B1* (Leu432Val)	Inuit	249	189 (76)	56 (23)	4 (1.6)	0.871	0.129	
	Europeans	262	89 (34)	131 (50)	42 (16)	0.590	0.410	0.0001
			Val/Val (wtI)	Val/Met	Met/Met	Val	Met	
*COMT* (Val158Met)	Inuit	252	44 (18)	124 (49)	84 (33)	0.421	0.579	
	Europeans	261	69 (26)	133 (51)	59 (23)	0.519	0.481	0.002

a p Value for comparison of the allele frequencies between Inuit and Europeans; no differences in allele frequencies were observed between genders (See Supplementary Table I for data).

The *CYP1A1* Val (variant) allele frequency was significantly higher in Inuit (0.460) compared to the Europeans (0.057) (p<0.0001) (Table III). The allele frequency of the variant *CYP1B1* (Val) also varied markedly amongst the two ethnic groups, with the allele frequency of 0.129 in Inuit versus 0.410 in Europeans (Table III). The difference in *COMT* genotype frequencies between Inuit and Europeans was not as marked as that of *CYP1A1* and *CYP1B1*. In Inuit, the frequency of the *COMT* Met allele (variant) was significantly higher (0.579) compared to the Europeans (0.481).

### Serum concentrations of CB-153 and p,p′-DDE in Inuit and Europeans and the relation to the genotypes

The serum concentrations of CB-153 and *p,p′*-DDE in our study population are given in Table IV. Inuit had 10 times higher serum CB-153, and 2 times higher *p,p′*-DDE levels than Europeans (p<0.0001). Inter-correlations between serum CB-153 and *p,p′*-DDE were observed with higher correlations in Greenland (r_s_=0.842; p<0.000) and Sweden (r_s_=0.747; p<0.0001), and lower correlations for the study groups of Poland and Ukraine (r_s_=0.277, p<0.005; and r_s_=0.446, p<0.0001, respectively).

**Table IV:** Serum levels of CB-153 and *p,p′*-DDE in the study population

		Europeans (Men)					Greenlandic Inuit (Men and women)					
			
		Poland	Sweden	Ukraine	All	p^a^	Men	Women	All	p^b^	p^c^	p^d^
CB-153 (ng/g lipid)	n	80	96	78	254		62	167	229			
	Median	16	210	47	48		298	581	503			
	Minimum	3	41	5	3		40	45	5			
	Maximum	129	1460	198	1460	<0.0001	5455	2298	5455	<0.0001	<0.0001	<0.0001
*p,p′*-DDE (ng/g lipid)	Median	571	236	881	528		704	1199	1075			
	Minimum	240	55	324	55		66	95	66			
	Maximum	2094	2251	11791	11791	<0.0001	13197	6438	13197	<0.0001	<0.0001	0.012

a p Value for comparison among the European countries

b p value for comparison between the sexes among Inuit

c p value for comparison between the Inuit and Europeans

d p value for comparison between Inuit and Europeans, men only.

In Table V, the serum concentrations of CB-153 and *p,p′*-DDE in the study population carrying different genotypes are given. Inuit carrying the *CYP1A1* Ile/Ile (wt) genotype had significantly lower adjusted serum CB-153 (p=0.008) and *p,p′*-DDE (p=0.061) than Inuit with at least one variant Val allele (Ile/Val *or* Val/Val). For the *CYP1B1*, the Inuit with Leu/Leu (wt) genotype had significantly higher serum CB-153 (p=0.029) than Inuit with at least one Val allele and higher serum *p,p′*-DDE concentrations (p=0.016) than Inuit with Leu/Val genotype. Pooling the Leu/Val and Val/Val genotypes (because of the few numbers of Val/Val carriers) showed that Inuit with at least one variant Val allele had significantly lower serum *p,p′*-DDE concentrations (p=0.004) (not shown). Furthermore, the Inuit carrying the *COMT* Val/Val (wt) genotype had significantly lower serum CB-153 (p=0.034) and *p,p′*-DDE concentrations (p=0.040), than Inuit with the Met/Met genotype.

**Table V:** Comparison of CB-153 and *p,p′*-DDE serum concentrations in persons with different genotypes (ANCOVA)

			CYP1A1			CYP1B1			COMT		
					
			Ile/Ile	Ile/Val	Val/Val	Leu/Leu	Leu/Val	Val/Val	Val/Val	Val/Met	Met/Met
Inuit	CB-153 (ng/g lipid)	n	60	118	51	171	53	3	39	109	80
		Median	435	532	602	567	380	403	391	508	596
		Adj mean^a^	5.92	6.22	6.19	6.20	5.92	6.03	5.92	6.12	6.25
		p^b^	0.008 (Ile/Ile different)			0.029 (Leu/Leu > Leu/Val)			0.034 (Val/Val < Met/Met)		
	*p,p′*-DDE (ng/g lipid)	n	60	118	51	171	53	3	39	109	80
		Median	963	1090	1287	1153	846	1703	880	1064	1287
		Adj mean^a^	6.73	7.05	6.94	7.00	6.70	7.33	6.71	6.93	7.07
		p^b^	0.061			0.016 (Leu/Leu > Leu/Val)			0.040 (Val/Val < Met/Met)		
Europeans	CB-153 (ng/g lipid)	n	196	26	0	87	125	42	64	131	58
		Median	60	44		33	74	86	76	45	56
		Adj mean^a^	4.04	3.95		3.92	4.05	4.08	4.09	3.98	4.05
		p^b^	0.571			0.421			0.647		
	*p,p′*-DDE (ng/g lipid)	n	196	26	0	87	125	42	64	131	58
		Median	475	555		597	483	428	516	498	524
		Adj mean^a^	6.15	6.20		6.32	6.19	6.06	6.33	6.18	6.14
		p^b^	0.741			0.181			0.287		

a Adjusted mean based on ln-transformed data.

b p Value for comparison of the CB-153 and *p,p′*-DDE between subjects with different genotypes using ANCOVA adjusting for sex, age, smoking, area of residence. Results for the pair wise comparison are given in the brackets (post hoc Bonferroni/Dunnett T3 test).

No differences were observed between the genotypes in Europeans and adjusted CB-153 and *p,p′*-DDE serum levels for any of the polymorphisms (Table V). Since the Swedish participants differed significantly from Ukraine and Poland participants in terms of *CYP1B1* genotype distribution, we compared the two POP proxy marker levels by genotypes excluding the Swedish participants from the European group and found no differences (data not shown).

Overall, multiple linear regression analysis (Supplementary Table II) showed the same results as the ANCOVA analysis (Table V).

## Discussion

About 88% of the Greenlandic population is of Inuit origin, and the population is relatively genetically homogenous. The Inuit originate from East Asia (2) and are therefore genetically different from Caucasians. Greenlandic Inuit are highly exposed to POPs through their traditional diet and it is important to consider the gene–environment interactions to elucidate possible health effects.

We report here, to our knowledge for the very first time, the allele and genotype distributions of *CYP1A1* Ile462Val (rs1048943), *CYP1B1* Leu432Val (rs1056836) and *COMT* Val158Met (rs4680) polymorphisms in Greenlandic Inuit, and compare the Inuit data with European Caucasians.

The allele and genotype frequencies of *CYP1A1* Ile462Val and *CYP1B1* Leu432Val polymorphisms are known to vary widely in different populations (40). In Inuit we found no significant genotype differences between the genders, which are in accordance with previous observations. Greenlandic Inuit had higher frequency of the variant *CYP1A1* Val allele (0.460) compared to Europeans, where the Val allele was rare (0.057). Our result on the prevalence of the Val allele in the European population is in accordance with the previous reported frequency of 0.052 for European populations (17), whereas the Val allele frequency in Inuit was much higher than the values reported for Asians (0.228) (17) but equal to frequencies in Nunavik Inuit (0.458) (41).

The polymorphic *CYP1B1* (Leu432Val) also varied between Greenlandic Inuit and Europeans, with lower frequency of the homozygous variant Val/Val genotype in the Inuit (1.6%) and higher in Europeans (16%). Our data for the Europeans are in accordance with the previous reported genotype and allele frequency distribution of Leu462Val polymorphism in Caucasians (24,42), and for the Greenlandic Inuit the distribution was similar to those reported for Asian populations (42).

*CYP1A1* and *CYP1B1* genes are inducible by some POPs and the enzymes are involved in the metabolism of a variety of environmental pollutants such as PAHs and endogenous substances such as estrogens. Since the conversion of both types of substrates has the potential to yield mutagenic and carcinogenic intermediates, polymorphisms in these genes might be a risk factor for the development of related malignancies. Taking into account that the *CYP1A1* Val variants result in increased catalytic activities of the enzyme that might increase the formation of carcinogenic agents such as ROS (7), it is of concern that Inuit having high levels of POPs have a higher frequency of the Val variants. It is feasible to consider the role of these gene polymorphisms in the susceptibility to carcinogenesis, depending on its combination with other genetic and environmental factors.

We also speculated whether the lower frequency of the *CYP1B1* variant Val allele in Inuit compared to Europeans might result in higher rate of activation of procarcinogenic compounds but the lower accumulation and exposure to the carcinogen 4-OH-E2 (21,43). However, future studies are needed to elucidate the role of *CYP1B1* polymorphism and contaminant interaction and the risk of hormone mediated cancers.

We analyzed possible differences between the serum levels of CB-153 and *p,p′*-DDE, as proxy marker for POP exposure, versus the investigated gene polymorphisms among the Inuit and European study population. We hypothesized that the studied polymorphisms in *CYP1A1* and *CYP1B1* might influence the serum POP levels. Among Inuit carrying the *CYP1A1* Ile/Ile (wt) genotype, lower median levels of CB-153 and *p,p′*-DDE were observed compared to those with at least one variant Val allele (Ile/Val or Val/Val), whereas among Europeans no significant differences were found in serum POP levels between *CYP1A1* genotypes. Considering the functional role of CYP1A1 in xenobiotic metabolizing, for example, PCBs (44) and the inducibility of *CYP1A1* by halogenated aromatic compounds (11), we did expect to find the opposite association, since the Val allele has higher enzyme activity and more likely to metabolize the PCBs to carcinogenic metabolites. However, the very high level of POPs in Inuit may have caused CYP1A1 enzyme substrate inhibition and therefore lower enzyme activities or co-presence of other environmental contaminants; for example, heavy metals could have consequently inhibited the CYP1A1 activity (45). Whether the serum level of the POPs is directly associated to *CYP1A1* polymorphisms is not known. Nevertheless, it is of concern that individuals carrying the higher inducible *CYP1A1* val variant also have higher serum POP levels and, therefore, might be more susceptible to POP-induced toxicities. However, further investigations are needed to elucidate whether there are any correlations between the high body burden of environmental contaminants, for example, POPs in Inuit, their higher *CYP1A1* Val variant allele frequency, and the risk for POP-mediated diseases.

For the *CYP1B1*, we observed that Inuit with Leu/Leu genotype (wt) had significantly higher serum CB-153 concentrations than Inuit with at least one variant Val allele. This association is not in agreement with the report that the CYP1B1 Leu432 form has slightly higher AHH activity towards procarcinogenic compounds than the Val432 variant (21) assuming a higher PCB conversion. The very high burden of POPs in Inuit might have influenced the AHH activity by substrate inhibition and can possibly explain the higher CB-153 level we observed in Inuit with Leu/Leu genotype. However, a higher AHH activity can also cause a higher reactive metabolite production and thus higher carcinogenicity. For Europeans, no differences were observed between the adjusted means of POP levels by genotypes. Another study on Caucasians from Slovakia found no statistically significant differences of total PCB levels between the *CYP1B1* genotypes (46). Since *CYP1B1* is also inducible by aromatic hydrocarbons, individuals with the high activity Val allele (22,23) and high levels of POPs might have increased risk for exposure to both environmental carcinogens and E2 metabolites.

The COMT gene that encodes the phase II enzyme inactivating the carcinogenic catechol estrogens (2-OH-E2 and 4-OH-E2), is also polymorphic and associated with ethnic differences, and the variant Met allele is shown to be associated with a 3–4 fold lower COMT enzyme activity (29,30). In this study, the frequency of the low activity Met allele in Inuit was higher (0.579) than that in Europeans (0.481). Our data for allele frequencies in Europeans were in agreement with the frequencies found in Caucasian controls (32). Whereas, the allele and genotype frequencies in Inuit differed significantly from those reported for Asians having a low frequency of the low activity variant Met allele (0.28) (32).

In a cohort of middle-aged men and in a cohort of early pubertal girls, the low-activity COMT (Met/Met) was associated with higher serum levels of estradiol than the other genotypes (47). Thus, the prevalence of homozygous *COMT* Met/Met variant is hypothesized to be associated with increased risk of developing estrogen-related cancers. Epidemiological studies have investigated the association of *COMT* polymorphism with cancers in hormone-responsive tissues, but the results are inconsistent (32,33).

Inuit carrying at least one variant Met allele encoding the COMT enzyme with lower activity had higher mean levels of CB-153 and *p,p′*-DDE compared to those with the Val/Val genotype. Several compounds such as catechol metabolites of PCBs are also potential substrates for the COMT enzyme and can inhibit COMT activity and thus the clearance of catechol estrogens (48). As for *CYP1A1* and *CYP1B1*, the expression of *COMT* gene can be modulated by exposure to environmental chemicals such as PCB and dioxins (49).

The observed correlations between the studied genotypes and POP marker levels should be viewed with caution, since the body levels of these contaminants are multifactorial dependent and, therefore, additional studies are needed to evaluate the relationship between concentration of these compounds in the blood and *CYP1A1, CYP1B1* polymorphism. Furthermore, polymorphisms in other genes involved in xenobiotic phase II detoxification such as glucuronyltransferase and sulphotransferase should be examined in future studies.

In summary, the present study showed ethnic-related differences in the frequency distribution in genetic polymorphisms of the *CYP1A1*, *CYP1B1* and *COMT* genes in Inuit and European populations. The prevalence of the *CYP1A1* variant Val allele was more frequent in Inuit than in Europeans, whereas the opposite was observed for the variant *CYP1B1* Val allele. For Inuit, we observed a significant association between CB-153 and *p,p′*-DDE serum levels and the gene polymorphisms in *CYP1A1*, *CYP1B1* and *COMT*, whereas no associations were found for Europeans. Information on the frequency distribution of metabolic gene polymorphisms can help in elucidating the role of these gene variants in the susceptibility of individuals towards environmental procarcinogens and the risk of certain diseases in different human populations.
